# The optimal screening interval for gastric cancer using esophago-gastro-duodenoscopy in Japan

**DOI:** 10.1186/1471-230X-12-144

**Published:** 2012-10-17

**Authors:** Daiki Kobayashi, Osamu Takahashi, Hiroko Arioka, Tsuguya Fukui

**Affiliations:** 1 Division of General Internal Medicine, Department of Internal Medicine, St. Luke’s International Hospital, Tokyo, Japan

## Abstract

**Background:**

Gastric cancer is one of the most significant diseases, and esophago-gastro-duodenoscopy (EGD) is one of screening methods for gastric cancer. This study was conducted to identify the optimal screening interval for gastric cancer using EGD in healthy adults.

**Methods:**

A retrospective cohort study was conducted on 3,723 healthy participants without a known diagnosis of gastric cancer at baseline from January 2005 to December 2010. Participants underwent annual health screenings, including EGD, at the Center for Preventive Medicine at St Luke’s International Hospital, a community teaching hospital in Japan. Participants with cytological abnormalities underwent further examination. A generalized estimating equation (GEE) was used to analyze the longitudinal data. We decided 0.5% of incidence of gastric cancer as a cutoff point for interval.

**Results:**

The mean age (SD) of the participants was 55 (11) years, and 1,879 (50.5%) were male. During the study period, gastric cancer was detected in 35 participants. However, the incidence varied based on their ages. In the age groups <40, 40–49, 50–59, 60–69 and ≥70 years old, the 5-year cumulative incidences (95%CI) of gastric cancer were 0% (0-0%), 0.3% (0.1-1.0%), 1.0% (0.5-1.8%), 1.4% (0.8-2.4%) and 1.9% (0.8-3.8%), respectively. The odds ratios of the incidence of gastric cancer per year, which were evaluated using GEE models for the age groups 40–49, 50–59, 60–69 and ≥70 years old, were 1.51 (95%CI: 0.91-2.49), 1.94 (95%CI: 1.31-2.86), 1.59 (95%CI: 1.23-2.06) and 1.46 (95%CI: 1.06-2.02), respectively.

**Conclusions:**

A screening for gastric cancer using EGD may be appropriate annually for healthy people over 70 years old, every two or three years for people 60–69 years old and every four years for people 50–59 years old. People younger than 50 years old may only need repeat screenings every five years or more.

## Background

Approximately 776,000 patients die from gastric cancer worldwide each year [1]. Japan has one of the highest rates of gastric cancer in the world; approximately 50,000 patients in Japan die from gastric cancer each year [2].

Some countries with high incidences of gastric cancer, including Japan [3], Chile [4] and Venezuela [5], recommend mass screening for gastric cancer, while other countries do not recommend routine screening [6]. In these high risk countries, mass screening for gastric cancer is expected to increase the early detection rate and reduce the mortality from gastric cancer based on previous large-scale case control studies and reviews [7,8].

Screening strategies for gastric cancer, however, are different among these high incidence countries. Esophago-gastro-duodenoscopy (EGD) is a screening method that is readily available and recommended by some experts in Japan [9]. Although there is a systematic review which recommends barium x-ray [10], the sensitivity of barium x-rays is lower than that of EGD. Considering the fact that approximately 40% of gastric cancers are still undetected [11], higher sensitivity of screening method is of paramount importance. Moreover, the incidence of complications during EGDs was reported as low as 0.012% and the mortality rate was only 0.001% in Japan [12]. Finally, EGD was shown to be cost effective in a systematic review [13].

However, the optimal interval of EGD screening for gastric cancer is yet to be determined. Therefore, we evaluated the optimal interval for EGD as a gastric cancer screening using a 5-year cohort.

## Methods

### Study participants

From January 2005 to December 2010, all participants (≥20 years old) who underwent EGD as a part of an annual health check-up program at the Center for Preventive Medicine at St. Luke’s International Hospital in Tokyo, Japan, were enrolled in this cohort study. We included all participants who have completed 5 years follow up. We also included patients who were found gastric cancer during study period, even if they have not completed the remaining years. We excluded people with a diagnosis of gastric cancer at baseline. Previous EGD was not restricted in our study.

### Data collection

We extracted data from the electronic health records of participants who had annual health screenings in 2005 (baseline year), 2006 (year 1), 2007 (year 2), 2008 (year 3), 2009 (year 4) and 2010 (year 5). The participants diagnosed with gastric cancer based on cytology were further evaluated at the Department of Gastroenterological Medicine or Digestive Surgery at St. Luke’s International Hospital. The cytology was evaluated by two pathologists independently. We aggregated information from the participants who were determined to have gastric cancer by cytology and were evaluated at the other hospitals. The St. Luke’s International Hospital’s ethics committee approved the protocol for this study.

### Measurements

The annual health check-up program contained an initial evaluation during which demographic and medical history information was collected. An EGD was performed by a gastroenterological specialist, and the cytological specimens were evaluated by two independent pathologists. Participants were divided into four age brackets: under 40 years of age, 40–49 years, 50–59 years, 60–69 years and 70 years or older. The incidence of gastric cancer, the odds ratio of the incidence over five years and the proportion of gastric cancers to the number of biopsies were used as indicators to define the optimal intervals for screening. An incidence of 0.5% for gastric cancer was considered the cutoff point based on a previous interval study and the Japanese prevalence of gastric cancer [14].

### Statistical methods

Data were analyzed using descriptive statistics, including the mean, variance, standard deviation (SD), and percentage. Chi-squared and Fisher’s exact tests were used for cross-tabulated data, and t-tests for continuous data. Additionally, generalized estimating equations (GEE) [15], which were adjusted for possible risk factors, such as age, gender, alcohol consumption, smoking, family history, past medical history of gastric ulcers, salt intake and body mass index (BMI) [16-19], were used to evaluate the odds ratio of the cumulative incidence of gastric cancer [20]. GEE is performed to estimate the parameters of a generalized linear model with a possible unknown correlation between outcomes [21]. With this analysis, we can estimate likelihood with considering correlation matrix for the vector of repeated observations from each subject [22].

The majority of the data analyses were performed using the SPSS software 15.0J (SPSS Japan, Tokyo, Japan), except for the 95% confidence intervals (CI) based on an exact binominal [23] and the GEE methods which were calculated using the Stata version 10 (STATA Corp., College Station, TX, USA).

## Results

From January 2005 to December 2010, 3,723 participants completed all five yearly health screenings. The mean age (SD) of participants was 55 (12) years, and 1,879 participants (50.5%) were male. The mean (SD) body mass index (BMI) was 22.5 (3.1) kg/m [2]. Of the participants, 685 (18.4) had a family history of gastric cancer, and 242 (6.5) had a past medical history of gastric cancer. Other baseline characteristics are shown in Table 1.

**Table 1:** Participant characteristics

	**Gastric cancer patients over five years (n=35)**	**Normal participants (n=3,688)**	**Total (n=3,723)**	**Mean difference (p- value)**
**Age, mean (SD)** years	62 (9)	55 (12)	55 (12)	<0.01
**Gender, male (%) n**	25 (71.4)	1,854 (50.3)	1,879 (50.5)	<0.01
**BMI, mean (SD)** kg/m [2]	23.8 (3.1)	22.5 (3.1)	22.5 (3.1)	0.01
**SBP** mean (SD), mmHg	125.4 (13.6)	120.7(18.2)	120.8 (18.2)	0.05
**DBP** mean (SD), mmHg	77.1 (7.9)	74.9 (11.3)	75.0 (11.3)	0.12
**Heart rate** mean (SD), bpm	69.5 (9.4)	73.4 (11.3)	73.3 (11.3)	0.04
**White blood cell** mean (SD), *10 [3] /μl	5.6 (1.5)	5.1 (1.4)	5.1 (1.4)	0.06
**Hemoglobin** mean (SD), g/dl	14.1 (1.0)	13.9 (1.3)	13.9 (1.3)	0.21
**Platelet** mean (SD), *10 [6] /μl	224.2 (43.5)	234.4 (51.0)	234.3 (51.0)	0.24
**Fasting blood sugar** mean (SD), mg/dl	108.3 (18.8)	101.5 (17.2)	101.6 (17.3)	0.39
**HgbA1c** mean (SD), %	5.3 (0.7)	5.2 (0.6)	5.2 (0.6)	0.27
**LDL-cho** mean (SD), mg/dl	117.0 (21.8)	120.5 (29.0)	120.5 (29.0)	0.47
**HDL-cho** mean (SD), mg/dl	58.2 (12.8)	62.8 (15.7)	62.7 (15.7)	0.08
**TG** mean (SD), mg/dl	107.4 (53.0)	102.8 (70.7)	102.8 (70.5)	0.70
**Family history of gastric cancer** n (%)	8 (1.2)	677 (18.4)	685 (18.4)	0.31
**Alcohol habit** n (%)	24 (68.6)	2,157 (58.5)	2,181 (58.6)	0.15
**Smoker: Brinckman index** n (SD)	430.7 (648.0)	210.6 (412.7)	21.7 (415.9)	0.05
**Salt intake**: g per day g (SD)	12.7 (3.4)	12.2 (3.8)	12.2 (3.8)	0.44
**Gastric ulcer** n (%)	3 (1.2)	239 (6.5)	242 (6.5)	0.40

Number of cigarettes smoked per day times the number of smoking years.

**Figure 1:**
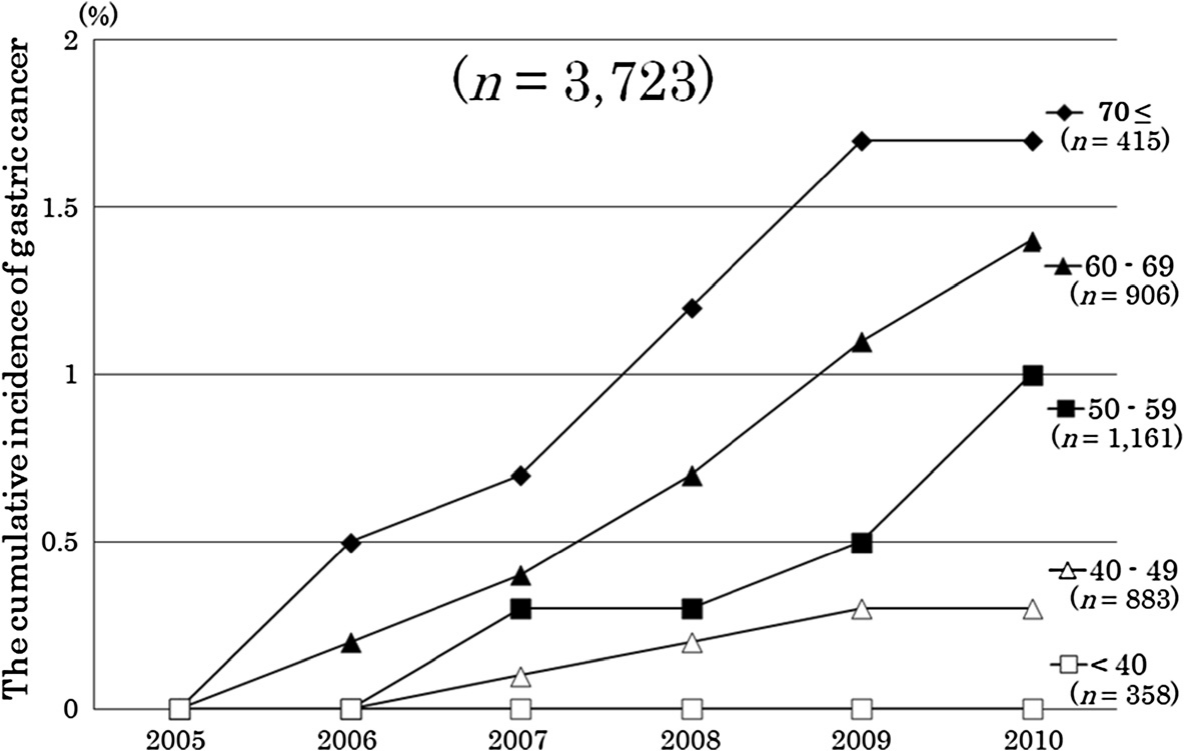
The cumulative incidence of gastric cancer for 5 years by age group.

After 5 years, the cumulative incidence of gastric cancer was 0.9% (95% CI, 0.7-1.3). However, the incidence varied greatly between age groups. At 5 years, the participants who were <40, 40–49, 50–59, 60–69 and ≥70 years old at baseline had cumulative incidences (95% CI) of gastric cancer of 0% (0-0%), 0.3% (0.1-1.0%), 1.0% (0.5-1.8%), 1.4% (0.8-2.4%) and 1.9% (0.8-3.8%), respectively (Figure 1).

During this 5 years period, 964 biopsies were performed, and 35 patients (3.6%, 95%CI: 2.5%-5.0%) were diagnosed with gastric cancer. All of them were followed throughout the study period (100%). Of these patients, 24 had adenocarcinoma, 6 had mucosa-associated lymphoid tissue (MALT) lymphoma, 4 had signet cell carcinoma, and 1 had carcinoid (Table 2). Among 24 patients with adenocarcinoma, 20 were able to be followed up with their stage of cancer. 17 patients were Stage IA, and 3 patients were Stage IB.

**Table 2:** Types of gastric cancer

	**<40**	**40-49**	**50-59**	**60-69**	**≥70**	**Total**
**Adenocarcinoma**	0	0	7	11	6	24
MALT^*^lymphoma	0	0	3	2	1	6
**Signet cell carcinoma**	0	3	1	0	0	4
**Carcinoid**	0	0	1	0	0	1
Total	0	3	12	13	7	35

MALT^*^: Mucosa-associated lymphoid tissue.

The patients with MALT lymphoma were treated by eradicating of *H. pylori*. Among other patients, 20 were determined to have cancer at an early stage and were treated with endoscopic submucosal dissection (ESD), and 9 underwent gastrectomy (Table 3).

**Table 3:** Types of treatment for gastric cancer

	**<40**	**40-49**	**50-59**	**60-69**	**≥70**	**Total**
ESD^*^	0	1	4	11	4	20
**Gastrectomy**	0	2	5	0	2	9
**H. pylori eradication**	0	0	3	2	1	6
**Total**	0	3	12	13	7	35

ESD^*^: Endoscopic submucosal dissection.

Table 4 shows the ratio of the incidence of gastric cancer per the number of biopsies. At 5 years, the participants who were <40, 40–49, 50–59, 60–69 and ≥70 years old at baseline had ratios (95% CI) of 0% (0-0%), 1.9% (0.4-5.4%), 3.5% (1.8-6.0%), 4.5% (2.4-7.6%) and 4.8% (2.0-9.7%), respectively. The ratio (95% CI) for all participants was 3.6 (2.5-5.0%).

**Table 4:** Positive biopsy ratio for gastric cancer over 5 years

	**<40**	**40-49**	**50-59**	**60-69**	**≥70**	**Total**
**2006**	0 / 4	0 / 27	0 / 55	2 / 57	2 / 18	4 / 161
**2007**	0 / 9	1 / 32	3 / 76	2 / 62	1 / 34	7 / 213
**2008**	0 / 4	1 / 35	1 / 70	2 / 53	2 / 26	6 / 188
**2009**	0 / 4	1 / 18	2 / 64	4 /67	2 / 35	9 / 188
**2010**	0 / 6	0 /47	6 / 79	3 /50	0 / 32	9 / 214
**Total (%)**	0 / 27 (0)	3 / 159 (1.9)	12 / 344 (3.5)	13 / 289 (4.5)	7 / 145 (4.8)	35 / 964 (3.6)

Incidence of gastric cancer / number of biopsies.

The odds ratios for the incidence of gastric cancer per year were estimated using GEE models adjusted for possible risk factors, including age, gender, alcohol consumption, smoking, family history, past medical history of gastric ulcers, salt intake and BMI. The independent odds ratios for the incidence of gastric cancer in the 40–49, 50–59, 60–69 and ≥70 years old age groups were 1.51 (95%CI: 0.91-2.49), 1.94 (95%CI: 1.31-2.86), 1.59 (95%CI: 1.23-2.06) and 1.46 (95%CI: 1.06-2.02), respectively. None of the participants who were <40 years old had gastric cancer. (Table 5) Although there was no statistical relationship between gastric cancer and other variables, high BMI tended to be associated with high incidence of gastric cancer.

**Table 5:** Odds ratios for the incidences of gastric cancer per year using a generalized estimating equation (GEE) in each age group, adjusted for age, gender, alcohol consumption, smoking, family history, past medical history of gastric ulcers, salt intake and body mass index (BMI)

**Age**	**Odds Ratio**	**95% Confidence Interval**	**Standard Deviation**	**p-value**
**<40**	No incidence			
**40-49**	1.51	0.91 - 2.49	0.36	0.11
**50-59**	1.94	1.31 – 2.86	0.38	<0.01
**60-69**	1.59	1.23 – 2.06	0.21	<0.01
**≥70**	1.46	1.06 – 2.02	0.24	0.02

## Discussion

This study showed that the incidence rates of gastric cancer significantly differed according to age. Accordingly, our recommended screening intervals were based on age brackets. For patients over 70 years old, screening for gastric cancer every year may be appropriate, and for patients 60–69 years old, every two or three years, for patients 50–59 years old, every three or four years, and for patients 40–49 years old and under 40 years old, every five years.

In the group of participants older than 70 years old, approximately 0.5% developed gastric cancer within the next year. The proportion of the incidence of gastric cancer to the number of biopsies was 7 / 145 (4.8%) compared to 35 / 964 (3.6%) in all participants. Having adjusted for multiple variables using GEE model, the odds ratio for the incidences of gastric cancer within the next year turned to be 1.46 (95%CI: 1.06-2.02). Cancer screening tends to be controversial in elderly people because of their remaining life expectancy. However, in Japan, the life expectancy is 80 years old for males and 86 years old for females [24], long enough for justifying cancer screening for people aged over 70 years.

For the 60–69 year-old age group, the incidence of gastric cancer exceeded 0.5% within the next three years. The proportion of the incidence of gastric cancer to the number of biopsies was 13 / 289 (4.5%), and the proportion was higher than for gastric cancer in all participants. In the GEE model, the odds ratio for the incidence of gastric cancer within the next year was 1.59 (95%CI: 1.23-2.06). Therefore, screening every two or three years may be appropriate.

For patients 50–59 years old, the incidence of gastric cancer was approximately 0.5% within the next four years. The proportion of the incidence of gastric cancer to the number of biopsies was 12 / 344 (3.5%), similar to the proportion of gastric cancer in all participants. Based on the GEE model, the odds ratio for the incidence of gastric cancer within the next year was 1.94 (95%CI: 1.31-2.86), and it was the highest among all the groups. Therefore, screening every four years may be reasonable.

For patients 40–49 years old, the incidence of gastric cancer was less than 0.5% within the next five years. The proportion of the incidence of gastric cancer to the number of biopsies was 3 / 159 (1.9%), and the proportion was lower than that for all participants. Based on the GEE model, the odds ratio for the incidence of gastric cancer in the next year was 1.51 (95%CI: 0.91-2.49), which attained no statistically significance. Therefore, screening every five or more years may be considered. However, in this group, all patients with gastric cancer had signet cell carcinoma, which has poor prognosis. This means that screening patients in this group may have to be considered carefully.

Among those under 40 years old, there were no patients with gastric cancer. Therefore, screening every five or more years may be appropriate for this age bracket.

In our study, 20 of 29 (69.0%, 95%CI: 49.2-84.7) patients with gastric cancer (excluding MALT lymphoma because of the differences in treatment) were detected in the early stage and treated with ESD. Compared to a previous study on barium x-ray screening program, the early stage detection rate in our study was high [25]. It is speculated that early detection of gastric cancer by nation which screening program in Japan has contributed to the reduced mortality from gastric cancer in the past decades [7]. In this regard, gastric cancer screening using EGD which detects gastric cancer in earlier stage than barium X-ray may be efficient.

In addition to that, early detection for gastric cancer was expected better prognosis than late detection. Previous study reported that the five years survival rate with gastric cancer in Stage I, II, III, IV were 99.1%, 72.6%, 45.9%, and 7.2% respectively in Japan [26]. Among 24 patients with adenocarcinoma, at least 20 patients were in Stage IA or IB in our study. Within them, 16 patients were treated by ESD, which was less invasive than gastrectomy. Therefore, patients in our study were expected not only early detection for gastric cancer, but also better prognosis.

There are some limitations to this study. First, this study was a retrospective cohort study. We could not determine the magnitude of false negative results. There is a report, however, which reported that the false negative rate for gastric cancer screening by EGD was nil while 12 months follow-up [27]. Therefore, we would think the effect of false negatives negligible in our study. Second, our data did not include information on *H. pylori*. It is said that 70-80% of people over 40 years old in Japan had *H. pylori [*[28]. A previous randomized controlled study showed that *H. pylori* eradication reduced precancerous lesions without affecting overall incidence of gastric cancer [29]. For this reason, the information on *H. pylori* infection is unlikely to affect our results from 5 years cohort study. Third, population in our study may have selection bias, because of the study design. However, the incidence of gastric cancer in our study was similar to that in the previous study in Japan [30]. Therefore, this is also unlikely to affect the result. Finally, our data didn’t have the information of atrophic gastritis. Previous study revealed that atrophic gastritis may be a major cause of gastric cancer [31]. For these reasons, additional studies are required to further evaluation.

## Conclusions

A screening program for gastric cancer may be appropriate every year for healthy people over 70 years old, every two or three years for those 60–69 years old and every four years for those 50–59 years old. People younger than 50 years old may only need screenings every five years or more.

## Competing interests

The authors declare that they have no competing pinterests.

## Authors' contributions

DK conducted this study, decided study design, analyzed data, and drafted the manuscript. OT contributed to study design decision, performing data analysis and making manuscript. HA checked study design, reviewed manuscript and contribute to discussion. TF organized this study and contributed to discussion. All authors read and approved the final manuscript."

## Pre-publication history

The pre-publication history for this paper can be accessed here:

http://www.biomedcentral.com/1471-230X/12/144/prepub

## References

[B1] World Health Organization The world health report 1996 Accessed Sep.16, 2011, at http://www.who.int/whr/1997/media_centre/50facts/en

[B2] Health, Labour and Welfare Ministry, Cancer research team Effectiveness evaluation based gastric cancer screening guideline 2006 Accessed Sep.16, 2011, at http://dcanscreen.ncc.go.jp/pdf/guideline/gastric_guide060714.pdf

[B3] Mizoue T Yoshimura T Tokui N Prospective study of screening for stomach cancer in Japan Int J Cancer 2003 106 103 107 10.1002/ijc.11183 12794764

[B4] Llorens P Gastric cancer mass survey in Chile Semin Surg Oncol 1991 7 339 343 10.1002/ssu.2980070604 1759081

[B5] Pisani P Oliver WE Parkin DM Alvarez N Vivas J Case–control study of gastric cancer screening in Venezuela Br J Cancer 1994 69 1102 1105 10.1038/bjc.1994.216 8198977 PMC1969457

[B6] Leung WK Wu MS Kakugawa Y Screening for gastric cancer in Asia: current evidence and practice Lancet Oncol 2008 9 279 287 10.1016/S1470-2045(08)70072-X 18308253

[B7] Hisamichi S Sugawara N Fukao A Effectiveness of gastric mass screening in Japan Cancer Detect Prev 1988 11 323 329 3390854

[B8] Fukao A Tsubono Y Tsuji I HI S Sugahara N Takano A The evaluation of screening for gastric cancer in Miyagi Prefecture, Japan: a population-based case–control study Int J Cancer 1995 60 45 48 10.1002/ijc.2910600106 7814150

[B9] Gastric carcinoma. In DynaMed [database online]. EBSCO Publishing http://web.ebscohost.com/dynamed/detail?sid=eec61ce4-ee17-4e2c-952b-2ea463997ac9%40sessionmgr10&vid=2&hid=18&bdata=JnNpdGU9ZHluYW1lZC1saXZlJnNjb3BlPXNpdGU%3d#db=dme&AN=116155. Updated November 19, 2011.Accessed November 28, 2011

[B10] Hamashima C Shibuya D Yamazaki H The Japanese guidelines for gastric cancer screening Jpn J Clin Oncol 2008 38 259 267 10.1093/jjco/hyn017 18344316

[B11] Japan Public Health Association Report of the Research Group for Evaluation of Effectiveness of Cancer Screening in Japan 1998

[B12] Kaneko EHH Kasugai T4th report of endoscopic complications: results of the Japan Gastroenterological Endoscopy Society survey 2004 Gastroenterol Endosc, Japanese 46

[B13] Dan YY So JB Yeoh KG Endoscopic screening for gastric cancer Clin Gastroenterol Hepatol 2006 4 709 716 10.1016/j.cgh.2006.03.025 16765306

[B14] Kobayashi D Takahashi O Fukui T Glasziou PP 2011 Optimal prostate-specific antigen screening interval for prostate cancer, Ann Oncol 10.1093/annonc/mdr413 21948815

[B15] Hanley JA Negassa A Edwardes MD Forrester JE Statistical analysis of correlated data using generalized estimating equations: an orientation Am J Epidemiol 2003 157 364 375 10.1093/aje/kwf215 12578807

[B16] Barstad B Sorensen TI Tjonneland A Intake of wine, beer and spirits and risk of gastric cancer Eur J Cancer Prev 2005 14 239 243 10.1097/00008469-200506000-00007 15901992

[B17] Doll R Peto R Boreham J Sutherland I Mortality from cancer in relation to smoking: 50 years observations on British doctors Br J Cancer 2005 92 426 429 15668706 10.1038/sj.bjc.6602359 PMC2362086

[B18] Tsugane S Sasazuki S Diet and the risk of gastric cancer: review of epidemiological evidence Gastric Cancer 2007 10 75 83 10.1007/s10120-007-0420-0 17577615

[B19] Yang P Zhou Y Chen B Overweight, obesity and gastric cancer risk: results from a meta-analysis of cohort studies Eur J Cancer 2009 45 2867 2873 10.1016/j.ejca.2009.04.019 19427197

[B20] Lipsitz SR Kim K Zhao L Analysis of repeated categorical data using generalized estimating equations Stat Med 1994 13 1149 1163 10.1002/sim.4780131106 8091041

[B21] Hardin J Hilbe J Generalized Estimating Equations 2003 Chapman and Hall/CRC, London

[B22] Ballinger G Using generalized estimating equations for longitudinal data analysis Organizational Research Methods 2004 7 127 150 10.1177/1094428104263672

[B23] Clopper C The use of confidence or fiducial limits illustrated in the case of the binomial Biometrika 1934 26 404 413 10.1093/biomet/26.4.404

[B24] World Health Organization. Japan Accessed Sep.16, 2011, at http://www.who.int/countries/jpn/en/

[B25] Kunisaki C Ishino J Nakajima S Outcomes of mass screening for gastric carcinoma Ann Surg Oncol 2006 13 221 228 10.1245/ASO.2006.04.028 16411143

[B26] Japanese Association of Clinical Cancer Centers Accessed Sep 12, 2012, at http://www.gunma-cc.jp/sarukihan/seizonritu/seizonritu.html

[B27] Vradelis S Maynard N Warren BF Keshav S Travis SP Quality control in upper gastrointestinal endoscopy: detection rates of gastric cancer in Oxford 2005–2008 Postgrad Med J 2011 87 335 339 10.1136/pgmj.2010.101832 21257996

[B28] Asaka M Kimura T Kudo M Relationship of Helicobacter pylori to serum pepsinogens in an asymptomatic Japanese population Gastroenterology 1992 102 760 766 1537513 10.1016/0016-5085(92)90156-s

[B29] Wong BC Lam SK Wong WM Helicobacter pylori eradication to prevent gastric cancer in a high-risk region of China: a randomized controlled trial JAMA 2004 291 187 194 10.1001/jama.291.2.187 14722144

[B30] Inoue M Tsugane S Epidemiology of gastric cancer in Japan Postgrad Med J 2005 81 419 424 10.1136/pgmj.2004.029330 15998815 PMC1743301

[B31] Derakhshan MH Malekzadeh R Watabe H Combination of gastric atrophy, reflux symptoms and histological subtype indicates two distinct aetiologies of gastric cardia cancer Gut 2008 57 298 305 10.1136/gut.2007.137364 17965056

